# Whole-genome sequencing identifies new genetic alterations in meningiomas

**DOI:** 10.18632/oncotarget.15043

**Published:** 2017-02-03

**Authors:** Mei Tang, Heng Wei, Lu Han, Jiaojiao Deng, Yuelong Wang, Meijia Yang, Yani Tang, Gang Guo, Liangxue Zhou, Aiping Tong

**Affiliations:** ^1^ The State Key Laboratory of Biotherapy and Cancer Center/Collaborative Innovation Center of Biotherapy, West China Hospital, West China Medical School, Sichuan University, Chengdu 610041, China; ^2^ College of Life Science, Sichuan University, Chengdu 610064, China; ^3^ Department of Neurosurgery, West China Hospital, West China Medical School, Sichuan University, Chengdu 610041, China

**Keywords:** whole-genome sequencing, meningioma, chromosome instability, copy number alteration, mutation

## Abstract

The major known genetic contributor to meningioma formation was *NF2*, which is disrupted by mutation or loss in about 50% of tumors. Besides *NF2*, several recurrent driver mutations were recently uncovered through next-generation sequencing. Here, we performed whole-genome sequencing across 7 tumor-normal pairs to identify somatic genetic alterations in meningioma. As a result, Chromatin regulators, including multiple histone members, histone-modifying enzymes and several epigenetic regulators, are the major category among all of the identified copy number variants and single nucleotide variants. Notably, all samples contained copy number variants in histone members. Recurrent chromosomal rearrangements were detected on chromosome 22q, 6p21-p22 and 1q21, and most of the histone copy number variants occurred in these regions. These results will help to define the genetic landscape of meningioma and facilitate more effective genomics-guided personalized therapy.

## INTRODUCTION

Meningiomas are the most common primary intracranial neoplasms in adults, accounting for 35.8% of all primary central nervous system (CNS) tumors diagnosed in the US [1, 2]. In China, meningiomas were the second most common CNS tumors, constituting 14.06% of all primary intracranial tumors [3]. While vast majority of meningiomas are grade I and do not invade the brain tissue, their growth within the intracranial space often leads to serious and potentially lethal consequences. Small percent higher-grade meningiomas (grades II and III) however, display malignant behavior characterized by brain invasion and higher recurrence rates [4, 5].

Previously, the only genetic driver of meningiomas to be identified was bi-allelic mutation or loss of the tumor suppressor gene neurofibromatosis 2 (*NF2*) on chromosome 22, encoding the protein Merlin. Loss of *NF2* is found in approximately 50% of sporadic meningiomas [6–9]. With the development of next-generation of sequencing, several recent studies have reported new driver mutations, including *TRAF7*, *KLF4*, *AKT1*, SMO, *PIK3CA*, *NOTCH2*, *SMARCB1*, *CHEK2*, *SMARCE1* and *POLR2A*, particularly in the remaining half of meningiomas with wild-type *NF2* [8, 9, 10, 11].

To discover more candidate mutations, here we used whole-genome sequencing approaches on a set of 7 primary unradiated grade I meningiomas and paired normal blood samples. As a result, most of the previously reported meningioma mutations (*NF2*, *TRAF7*, *NOTCH2*, *SMARCB1*, *CHEK2* and *AKT1*) were also detected in this study. Especially, we identified many novel mutations and copy number variants in meningioma.

## RESULTS

### Landscape of somatic alterations in meningioma

**Figure 1:**
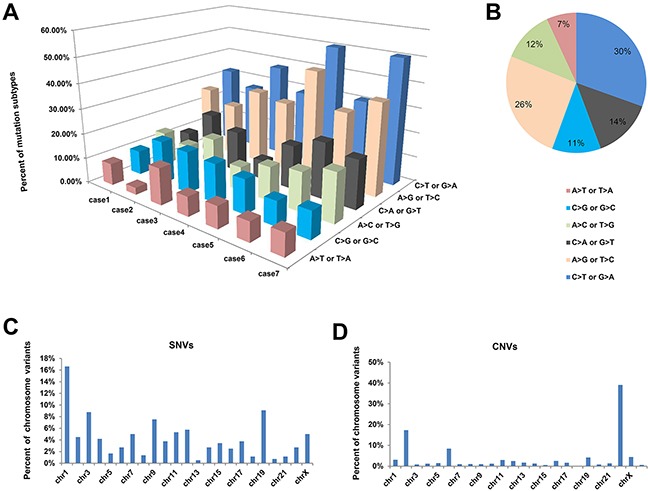
High frequency of C>T transversions identified in SNVs **A**. Histogram of mutation frequencies across all seven paired samples. Base substitutions were divided into six categories to represent the six possible base changes (each category was represented by a different color). **B**. Inset pie charts indicate the average distribution of substitutions. **C, D**. show the percentage of single nucleotide variants (SNVs) and copy number variants (CNVs) occurred on each chromosome. Chr means chromosome.

A total of 393,678 somatic single nucleotide variants (SNVs) were identified through whole-genome sequencing of the seven paired meningioma samples, including 103,289 inserted and deleted sequences (indels) and 290,389 single nucleotide polymorphisms (SNPs) (Supplementary Table 1 and 2). Among these SNVs, 1,338 somatic mutations caused changes in amino acid coding, including 1,284 SNPs (788 nonsynonymous, 411 synonymous, 18 stop-gain, 1 stop-loss and 32 splice site mutations) and 54 indels (13 frameshift deletion, 7 frameshift insertion, 18 nonframeshift deletion, 14 nonframeshift insertion and 1 stop-loss) (Supplementary Table 1 and 2). Of these SNVs, a high prevalence of C>T (equivalent G>A transversions on the complementary strand) base transversions were observed, comprising an average of 30.45% of the total substitutions (Figure 1A, 1B and Supplementary Table 3). Through analyzing the distribution of somatic mutations across individual chromosome, SNVs were more commonly found on chromosome 1, 3, 9 and 19, while chromosome 22, 2 and 6 carried more copy number variants (CNVs) (Figure 1C, 1D). Totally, 4,281 CNVs were identified in case 7 and the remaining six cases carried a total of 3,357 CNVs (Supplementary Table 4).

### Chromosomal rearrangements and genomic instability

**Figure 2:**
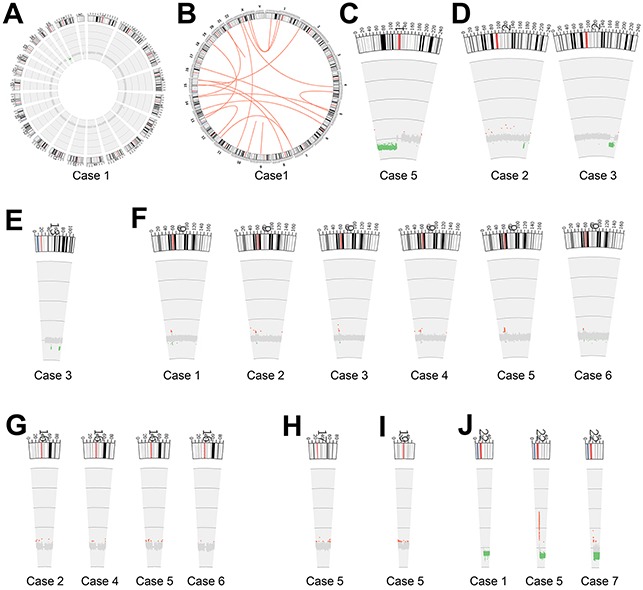
Patterns of chromosomal rearrangements and copy number alteration **A, B**. Representative Circos images show (A) copy-number alternations and (B) intra- or inter- chromosomal rearrangements (case 5) across the whole genome. Red lines refer to translocations and inversions. **C-J**. show large fragment alterations and regional copy number variants on each chromosome across seven meningioma samples. Green color represents copy number deletion, and red represents copy number amplification.

As shown in Figure 2, chromosomal rearrangements, including large fragment deletions and amplifications, inter- and intro- chromosomal translocations, were frequently identified across the seven paired samples. Representative chromosomal rearrangement maps were shown in Figure 2A (deletion on chromosome 22q) and Figure 2B (inter- and intro chromosomal translocations). We examined somatic rearrangements and in-frame gene fusions to identify potential fusion-gene products. As a result, a total of 99 rearrangements were identified, but no recurrent gene fusions were detected (Supplementary Table 4). Large fragment deletions occurred mainly on chromosome 1p, 2q33-q35 and 22q (Figure 2C, 2D & 2J). Loss of chromosome 22q was detected in three cases. Chromosome 22q contains several known tumor suppressors, such as *NF2*, *CHEK2 and SMARCB1*, and all the three tumor suppressors were deleted in the three cases (Table 1). Notably, small recurrent regional amplifications were identified on chromosome 6p21-p22 and 16p13 (Figure 2F, 2G). We also detected small regional amplifications on chromosomal 13q33, 17 and 19 (Figure 2E, 2H & 2I). We summarized these chromosomal arrangements and candidate genes harbored in these regions in Table 1. As shown, several chromatin regulators and multiple histone members (also listed in Supplementary Table 6) are very distinguishable.

**Table 1:** Variants of large chromosomal fragments and candidate genes located in these regions

CNVs	Chrom^a^	Case	Cytoband	Candidate genes	Histone members
**Amp^b^**	**Chrom 1**	3#	1q21.2	□	*HIST2H2AC*
		5#	1q21.2		*HIST2H3C, HIST2H4B*
					*HIST2H3A, HIST2H4A*
	**Chrom 6**	1#	6p22.1		*HIST1H2BL*
		2#	6p22.1-21.33	*CCHCR1*	
		3#	6p22.1-21.33	*DAXX*	*HIST1H4A*
				*FOXQ1*	*HIST1H1B, HIST1H4J*
		4#	6p22.1-21.33	*MRPS18B, SLC44A4*	*HIST1H2BE*
		5#	6p22.1-21.33	*SLC44A4*	*HIST1H2AD*
				*DDX39B*	*HIST1H2BF*
				*DDR1*	*HIST1H2AJ*
		6#	6p22.1-21.33	*CCHCR1*	*HIST1H2AL*
				*SLC44A4*	*HIST1H2AK*
				*NOTCH4*	*HIST1H3I, HIST1H1A*
	**Chrom 16**	2#	16p13.3, 16p13.11 & 16p13	*LOC100288162*	
		4#	16p13.3, 16p13.11 & 16p13	*LOC100288162*	
		5#	16p13.3, 16p13.11 & 16p13	*LOC100288162*	
		6#	16p13.3, 16p13.11 & 16p13	*LOC100288162*	
	**Chrom 22**	5#	22q11.1 & 22q11.21	*BCL2L13*	
**Del^c^**	**Chrom 1**	1#	1q21.2		*HIST2H2BC, HIST2H3D*
		5#	1p13.2-36.23	*HDAC1, KDM1A*,	
				*KDM4A, CSF1*,	
				*ARID1A*	
		7#	1q21.2		*HIST1H1A-HIST1H2BG (13 members)*
	**Chrom 2**	2#	2q35	*IGFBP5*	
				*SMARCAL1*	
		3#	2q33.1-35	*CASP8, CASP10*,	
				*BARD1, IGFBP5*,	
				*SMARCAL1*	
	**Chrom 6**	1#	6p22.1-21.33	*TRIM26*	*HIST1H3A*
				*CCHCR1, MDC1*	*HIST1H2APS1*
		2#	6p22.2		*HIST1H4E*, *HIST1H2AG*
		4#	6p22.2		*HIST1H2BN*
		7#	6p22.2		*HIST2H2AA3-HIST3H3 (48 members)*
	**Chrom 13**	3#	13q33.1-33.3	*EFNB2*	
	**Chrom 22**	1#	22q11.21-13.33	*NF2, BID, BIK, CHEK2, SMARCB1*	
		5#	22q11.21-13.33	*NF2, BIK, SMARCB1, CHEK2*	
		7#	22q11.21-13.33	*NF2, BID, BIK, SMARCB1, CHEK2*	

Chrom^a^ means Chromosomes. Amp^b^, Amplification. Del^c^, Deletion

### Candidate mutations and network analysis

**Figure 3:**
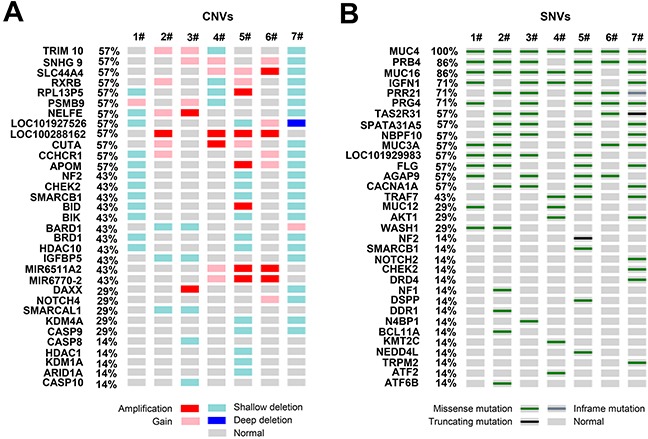
Recurrently altered genes and selected candidate mutations The most frequently altered genes and selected candidate mutations were shown. Mutation subtypes are denoted by color. If multiple mutations were found in a gene in a single sample, only one is shown. Maps were generated by using cBioPotal tools. Deep deletion (with copy-number level ranging from 0 to 0.49); Shallow deletion (0.5-1.5 range).

**Figure 4:**
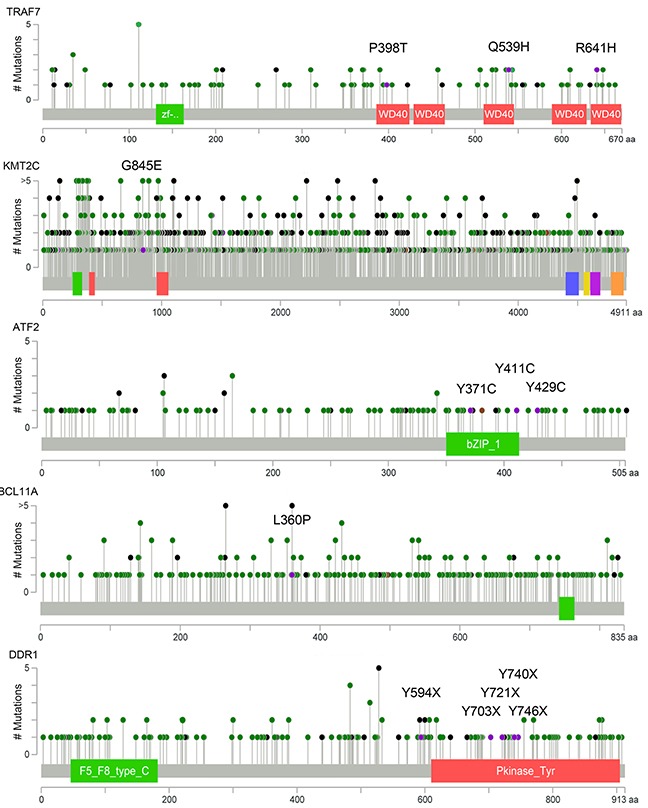
Novel mutations identified in five representative candidate genes Schematic mutation maps of five representative candidate genes (*TRAF7*, *KMT2C*, *ATF2*, *BCL11A* and *DDR1*) across multiple human tumor types were generated by using cBioPotal tools. Green dots refer to missense mutation and dark dots represent truncation. Purple dots with annotations show mutations identified in this study.

**Figure 5:**
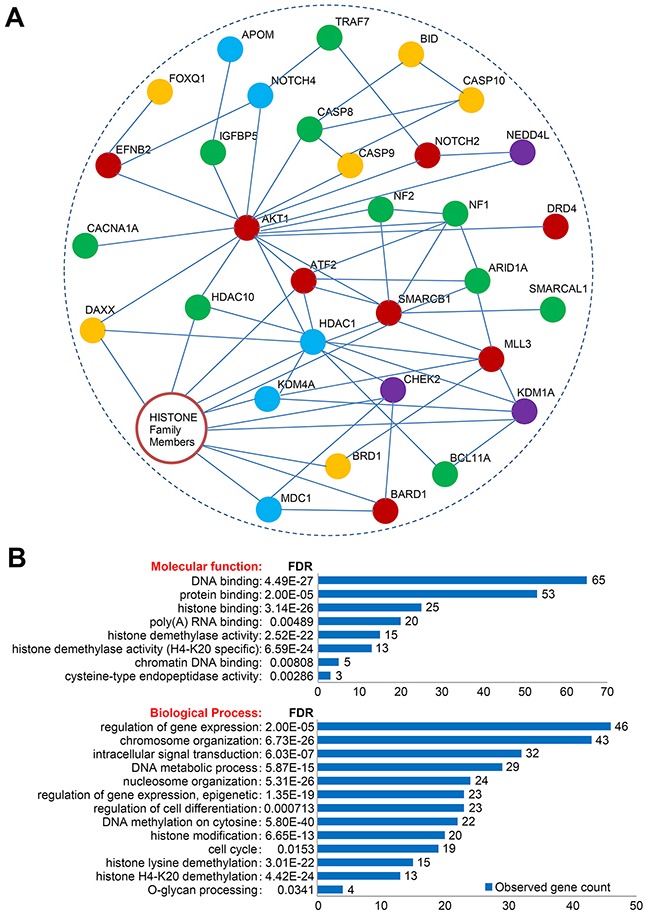
Network and Gene Ontology/pathway analysis of candidate genes **A**. A total of 130 selected candidate genes were subjected to interaction network analysis using STRING website tools. All protein-protein interactions were manually drawn based on the analysis results of STRING. The remaining proteins without significant interaction with this main network were not shown. **B**. Gene Ontology/pathway analysis of the candidate genes. Only top enriched GO categories were shown. Gene list can be found in Supplementary Table 8. FDR, false discovery rate.

Figure 3A shows an overview of the recurrently altered genes and candidate mutations. As shown, deletions in multiple functionally important genes (such as *HDAC1, HDAC10, KDM4A, KDM1A, SMARCAL1, ARID1A, BIK, BRD1, CASP8, CASP9* and *CASP10*) were observed. Notably, three non-coding RNA (*LOC100288162, MIR6511A2* and *MIR6770-2*) were recurrently amplified. Of the 1,341 coding-changing mutations, several known meningioma-driver mutations (*NF2*, *TRAF7*, *NOTCH2*, *SMARCB1*, *CHEK2* and *AKT1*) and a handful of novel candidate genes (such as *BCL11A*, *ATF2*, *DDR1*, *N4BP1*, *ATF6B*, *DSPP*, *NEDD4L*, *DRD4*, *TRPM2* and *KMT2C*) were identified (Figure 3B). As shown, three cases harbored *TRAF7* mutations (the mutation sites are different from previously reported) and two known meningioma-driver mutations (*AKT1*^E17K^ and *SMARCB1*^R377H^) were also detected. All the seven cases harbored mutations in *MUC4* and six cases carried mutations in *MUC16*, however the mutation sites are almost different from each other (Supplementary Table 7). Schematic mutation maps of five representative candidate genes were shown in Figure 4, and binary alignment (BAM) map of a representative somatic mutation in *TRAF7* was shown in Supplementary Figure 1. When subjected these recurrent alterations and candidate mutations (Supplementary Table 8) to network analysis, the most prominent alterations are involved in chromatin regulation (Figure 5A). DNA binding and chromosome organization rank near the top of the GO molecular function and biological processes categories respectively (Figure 5B).

### Overview of candidate mutations across the seven paired meningioma samples

As shown in Table 2, three cases (case 1, 3 & 7) carried loss of chromosome 22 and deletions of three known meningioma-driver genes (*NF2*, *CHEK2* and *SMARCB1*). Of the three cases, case 5 also harbored a truncation mutation in *NF2* and a mutation in *SMARCB1*. Case 7 co-occurred mutations in *CHEK2*, *NOTCH2*, *TRAF7* and *AKT1*. Case 4 carried copy number alterations in two histone members, as well as candidate mutations in *TRAF7*, *AKT1*, *KMT2C* and *ATF2*.

**Table 2:** Overview of candidate mutations across seven paired meningioma samples

	Case 1	Case 2	Case 3	Case 4	Case 5	Case 6	Case 7
**Chrom loss^a^**							
Chrom 1					Loss		
Chrom 2			Loss				
Chrom 22	Loss				Loss		Loss
**CNVs**							
*NF2*	Del^b^				Del		Del
*SMARCB1*	Del				Del		Del
*CHECK2*	Del				Del		Del
Others^e^	*BID, BIK*, *HDAC10*, *BRD1*	*BARD1, SMARCAL1*	*BARD1, SMARCAL1, CASP8, CASP10, DAXX*		*BID, BIK, HDAC10, BRD1, HDAC1, ARID1A*, *KDM1A, KDM4A*	*NOTCH4*	*BID, BIK, HDAC10, BRD1*, *BARD1*, *DAXX*
**SNVs**							
*NF2*					Trunc^c^		
*SMARCB1*					Mut^d^		
*CHECK2*							Mut
*NOTCH2*							Mut
*TRAF7*				Mut	Mut		Mut
*AKT1*				Mut			Mut
Others^e^		*DDR1, NF1, BCL11A, ATF6B*	*N4BP1*	*KMT2C, ATF2*	*NEDD4L*		*DRD4, CHECK2*

Chrom loss^a^, Del^b^, Trunc^c^, and Mut^d^ mean loss of chromosome fragments, deletion, truncation and mutation respectively. *NF2, SMARCB1, CHECK2, NOTCH2, TRAF7 and AKT1* are known meningioma-driver mutations. Other^e^ are candidates identified in this study.

Of the remaining three *NF2* wild-type meningiomas which also lack other known meningioma-driver mutations: i) Case 2 contained deletions of *BARD1*, *SMARCAL1* and two histone members, as well as mutations in *NF1*, *DDR1*, *BCL11A* and *ATF6B*; ii) Case 3 harbored deletions on Chromosome 2q containing *CASP8*, *CASP10*, *BARD1* and *SMARCAL1*, and amplifications on Chromosome 6p containing *DAXX* and three histone members; iii) Case 6 harbored amplifications on Chromosome 6p containing *NOTCH4* and four histone members.

## DISCUSSION

In this study, numerous SNVs and CNVs have been identified by whole-genome sequencing. Of these SNVs, a high prevalence of C>T base transversions were observed. Different cancer types have different mutational signatures. For example, Lung adenocarcinoma and lung squamous cell carcinoma contain increased C>A transversions [12], while microsatellite unstable gastric cancer were observed to have a higher mutation prevalence of both C>T transitions and C>A transversions [13]. It has been reported that clustered C>T mutation at CpG sites was associated with aberrant DNA methylation and gene expression regulation [14]. Thus further studies to explore the clustered C>T mutation status at CpG sites and their potential functions are needed.

Many candidates, not previously implicated in meningioma, have been uncovered in the current analysis. Of the identified somatic mutations, it is interesting that mutations in several mucin members, including *MUC4*, *MUC12, MUC16* and *MUC3A*, were frequently observed. Mucins are a family (over 20 members) of high molecular weight, heavily glycosylated proteins produced by epithelial tissues. Increased mucin expression, especially *MUC1* and *MUC4*, occurs in many adenocarcinomas [15]. *MUC16*, also known as carcinoma antigen 125, is a prominent biomarker of ovarian cancer. Next-generation sequencing also found frequent mutations in several *MUCIN* family genes in a variety of cancer types [16]. In the present study, although frequently mutated, the mutation amino acid sites are different from each other in the sequenced samples. It is interesting to know why *MUCINs* mutated so frequently and their functions in the genesis and development of meningioma.

Notably, we identified recurrent mutations or alterations in multiple chromatin regulators, including multiple histone members, histone-modifying enzymes (*HDAC1, HDAC10, KMT2C, KDM4A* and *KDM1A*), and other chromatin regulators (*SMARCB1, SMARCAL1, DDR1, DAXX, MDC1* and *ARID1A*). These chromatin regulators are the major category among all the identified alterations. AR42, a novel histone deacetylase inhibitor, has been reported to be a candidate drug for vestibular schwannoma and meningioma [17]. So it might be a hopeful approach to develop drugs targeting chromatin regulators for meningioma therapy.

It is known, alterations in chromatin regulators can result in chromosome instability [18]. In this study, large fragment deletions were detected on the long arms of chromosome 22 & 2, as well as the short arm of chromosome 1. Small regional copy number variants were observed on chromosome 6, 16, 17 and 19. Particularly interesting phenomenon is that, 6 cases harbored recurrent amplifications on chromosome 6p21-p22, and 4 cases contained amplifications on chromosome 16p13. We found most of the identified histone members are located on 6p21-p22, and three non-coding RNAs located on 16p13 were also recurrently amplified. So it is very interesting to know whether these recurrent genetic alterations are potential key drivers for meningioma.

In summary, we performed whole-genome sequencing across seven meningioma cases. Three cases harbored loss of chromosome 22q and *NF2*. The remaining four *NF2* wild-type meningioma cases were found to carry multiple known and novel somatic mutations. Particularly, we identified recurrent alterations in multiple chromatin regulators, which constitute the major category of the identified alterations. Notably, all samples harbored copy number variants in histone members, and most of them were identified on chromosome 22q, 6p21-p22 and 1q21. These data provide useful clues for the development of new therapeutic approaches against meningioma.

## MATERIALS AND METHODS

### Sample collection and DNA extraction

This study was approved by the Institutional Review Board (IRB) of the West China Hospital (File No. SKLB20140830-02), Sichuan, China, and informed consent were obtained from all the parents. All experiments were performed in accordance with relevant guidelines. All samples were fresh-frozen primary resections from individuals who were newly diagnosed as meningioma and hadn't treated with chemotherapy or radiation previously (Supplementary Table 5). Genomic DNA was extracted from freshly isolated tissues and blood samples with QIAamp DNA Mini kits (Qiagen). DNA concentrations were measured with NanoDrop 2000 (Thermo Fisher Scientific).

### Whole genome sequencing and data analysis

Library construction (5 μg DNA), whole-genome sequencing and data analysis were carried out by WuXi AppTec, China. Briefly, DNA was sheared with Covaris S220 Sonicator (Covaris) to a target of 300–400 bp average size. Fragmented DNA was purified using Sample Purification Beads (Illumina). Adapter-ligated libraries were prepared with the TruSeq Nano DNA Sample Prep Kits (Illumina) according to Illumina-provided protocol. DNA concentrations of the resulting sequencing libraries were measured with the Qubit 2.0 fluorometer dsDNA HS Assay (Thermo Fisher Scientific). Quantities and sizes of the resulting sequencing libraries were analyzed using Agilent BioAnalyzer 2100 (Agilent). The libraries were used in cluster formation on an Illumina cBOT cluster generation system with HiSeq X HD PE Cluster Kits (illumina). Paired-end sequencing was performed by using an Illumina HiSeq X™ Ten following Illumina-provided protocols for 2×150 paired-end sequencing (∼30x coverage, ∼90 Gb raw data /sample, (PE150, Q30 ≥ 80%)). Data quality control, alignment with UCSC hg19, variant (CNV, Indel, SNV and SV) calling, annotation, statistics and filter were performed by WuXi AppTec with public tools and databases including MuTect, VarScan2, GATK, CGATools, IGV, Circos, SAMtools, Polyphen2, SIFT, DBSNP, 1000genomes and COSMIC as previously reported [19].

### Data visualization and network analysis

Visualization of the distribution of different CNVs/SNVs subtypes were performed by using OncoPrinter tools from cBioPortal (http://www.cbioportal.org/). Mutation profiles of a single gene across various tumor types were also generated via MutationMapper tools from cBioPortal. Gene interaction network and Gene Ontology (GO) enrichment were analyzed by STRING online tools (http://string-db.org/).

## SUPPLEMENTARY FIGURE AND TABLES





## Abbreviations

SNVs: Single Nucleotide Variants;
CNVs: Copy Number Variants;
SVs: structural variations
SNPs: Single Nucleotide Polymorphisms;
Indel: Insertion and Deletion;
