# Supplementary material for: Whole-genome sequencing identifies new genetic alterations in meningiomas
Source: Oncotarget. 2017 Feb 3;8(10):17070–80. doi: 10.18632/oncotarget.15043 (PMC5370023; doi:10.18632/oncotarget.15043)
Supplement: Supplementary file 1 [file oncotarget-08-17070-s001.pdf]

Whole-genome sequencing identifies new genetic alterations in meningiomas

SUPPLEMENTARY FIGURE AND TABLES

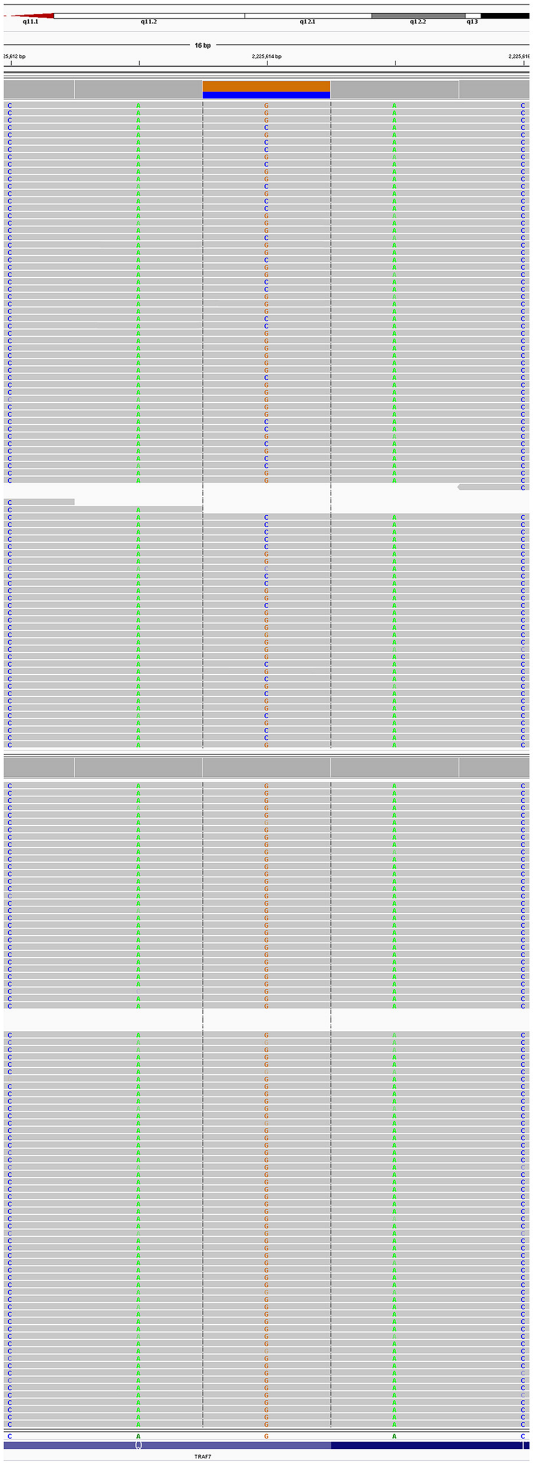

Supplementary Figure 1: Binary alignment map of TRAF7 c.1617G>C (Q539H) mutation.

Supplementary Table 1: Summary of identified insertions and deletions (Indels)

	case 1	case 2	case 3	case 4	case 5	case 6	case 7	total
**Total Indel number**	11680	11428	12375	11598	12577	19100	24531	103289
**exonic[a]**	7	5	7	10	6	11	8	54
**frameshift deletion**	1		2	5	1	1	3	13
**frameshift insertion**		2		3	1		1	7
**nonframeshift deletion**	4	2	3	1	1	4	3	18
**nonframeshift insertion**	2	1	2	1	2	5	1	14
**Stoploss[b]**						1		1
**unknown**					1			1
**Intergenic[c]**	6455	6362	6889	6360	6988	10231	13326	56611
**Intronic[d]**	4308	4161	4487	4277	4596	7310	9240	38379
**ncRNA_intronic**	618	606	671	680	707	1090	1338	5710
**ncRNA_exonic**	25	17	30	25	22	38	51	208
**Downstream[e]**	116	88	114	71	93	160	178	820
**Upstream[f]**	80	98	85	94	71	128	202	758

a, Indels happened within exons.
b, lead to loss of the normal stop codon by Indels.
c, regions between genes.
d, within introns.
e, f, refer to a relative position downstream or upstream of the transcription start site.

Supplementary Table 2: Summary of the identified single nucleotide polymorphisms (SNPs)

Variant statistics items	Case 1	Case 2	Case 3	Case 4	Case 5	Case 6	Case 7	Total number
**Total SNPs number**	34231	46867	39534	35750	38954	45261	49792	290389
**exonic**	153	198	212	135	204	164	218	1284
**nonsynonymous SNV**[a]	95	121	122	84	133	107	126	788
**synonymous SNV**[b]	47	68	70	42	65	44	75	411
**splicing**[c]	2	4	5	4	6	7	4	32
**stopgain**[d]		5	2		2	5	4	18
**unknown**	10	4	18	9	4	8	13	66
**stoploss**[e]	1							1
**intergenic**[f]	20450	26917	23363	21386	23189	26866	29822	171993
**intronic**[g]	10445	15562	12435	11078	12122	14401	15415	91458
**ncRNA_intronic**[h]	2201	2933	2423	2184	2394	2748	3136	18019
**ncRNA_exonic**[i]	202	171	176	159	189	177	182	1256
**upstream**[j]	303	421	355	316	326	317	390	2428
**downstream**[k]	244	310	272	262	256	293	325	1962
**UTR3**[l]	184	279	228	173	219	251	252	1586
**UTR5**[m]	40	56	51	40	41	30	36	294
**upstream;downstream**	5	16	14	11	8	6	12	72
**ncRNA_splicing**	2			2		1		5

a, SNVs, Single Nucleotide Variants; A nonsynonymous SNV is a nucleotide mutation that results in a change in amino acid.
b, synonymous SNV is a nucleotide substitution which do not alter the amino acid sequence.
c, SNPs that occur in splicing site.
d, SNPs that result in a stop codon.
e, lead to loss of the normal stop codon.
f, regions between genes
h, i, ncRNA, non-coding RNA.
j, k, refer to a relative position upstream or downstream of the transcription start site.
l, m, 3’, 5’ un-translational region.

Supplementary Table 3: Summary of base transversions

	C>A or G>T*	C>G or G>C	A>T or T>A	A>C or T>G	A>G or T>C	C>T or G>A
**case 1**	9.71%	9.71%	8.74%	13.59%	26.21%	32.04%
**case 2**	20.71%	17.14%	2.86%	11.43%	21.43%	26.43%
**case 3**	16.50%	16.50%	14.56%	17.48%	30.10%	37.86%
**case 4**	7.77%	15.53%	7.77%	9.71%	28.16%	29.13%
**case 5**	17.48%	13.59%	8.74%	13.59%	43.69%	50.49%
**case 6**	22.33%	9.71%	7.77%	15.53%	30.10%	31.07%
**case 7**	19.42%	10.68%	8.74%	19.42%	36.89%	50.49%
**average**	13.84%	11.30%	6.87%	11.96%	25.58%	30.45%

*, Base transverstions occur both in sense and antisense strands. If C>A mutation occur in sense strand, antisense strand will harbor a G>T mutation.

Supplementary Table 4: Summary of the identified CNVs and SVs

	CNVs	SVs
**case 1**	688	20
**case 2**	129	13
**case 3**	288	26
**case 4**	103	15
**case 5**	2009	25
**case 6**	140	0
**case 7**	4281	0
**total**	7638	99

CNVs, Copy-number variations
SVs, structural variations

Supplementary Table 5: Clinical characteristics of the seven pared meningioma samples

Sample number	WHO grade	Tumor type[a]	Radiated[b]	Age	Gender[c]	Histology	Location
**Case 1**	Grade I	P	N	61	F	Fibroblastic	Left Frontal/Convexity
**Case 2**	Grade I	P	N	46	F	Meningothelial	Left Frontal/Convexity
**Case 3**	Grade I	P	N	42	F	Fibroblastic	Left Frontal/Convexity
**Case 4**	Grade I	P	N	50	F	Transitional	Right Frontal/Convexity
**Case 5**	Grade I	P	N	48	F	Transitional	Left Frontal/Convexity
**Case 6**	Grade I	P	N	51	M	Meningothelial	Right frontal/Convexity
**Case 7**	Grade I	P	N	43	F	Fibroblastic-Transitional	Right parasagittal/Falcotentorial

a, P, primary tumor
b, N, No
c, F, female; M, male

Supplementary Table 6: CNVs in histone members identified in the seven pared meningioma samples.

See Supplementary File 1

Supplementary Table 7: Nonsynonymous SNVs in muncin members identified in the seven pared meningioma samples

Genes	Case #	Chromosome	Position	cDNA change	AA Change[a]
**MUC4**	case 1	3q29	195508163	c.A10288C	p.T3430P
**MUC4**	case 2	3q29	195513031	c.T5420C	p.I1807T
**MUC4**	case 3	3q29	195509030	c.A9421G	p.S3141G
**MUC4**	case 4	3q29	195513611	c.A4840G	p.T1614A
**MUC4**	case 5	3q29	195509075	c.A9376G	p.T3126A
**MUC4**	case 6	3q29	195507323	c.A11128G	p.T3710A
**MUC4**	case 7	3q29	195508115	c.A10336C	p.T3446P
**MUC16**	case 1	19p13.2	8999560	c.A40615G	p.K13539E
**MUC16**	case 2	19p13.2	8999439	c.C40736T	p.T13579I
**MUC16**	case 3	19p13.2	9021112	c.A37211C	p.D12404A
**MUC16**	case 4	19p13.2	9024481	c.G37052A	p.S12351N
**MUC16**	case 5	19p13.2	8993426	c.G41663A	p.S13888N
**MUC16**	case 7	19p13.2	9012490	c.G38716A	p.G12906S
**MUC3A**	case 1	7q22.1	100550552	c.A1133T	p.E378V
**MUC3A**	case 2	7q22.1	100550224	c.G805A	p.V269M
**MUC3A**	case 6	7q22.1	100551106	c.A1687G	p.K563E
**MUC3A**	case 7	7q22.1	100550432	c.C1013T	p.T338I
**MUC12**	case 1	7q22.1	100647338	c.C13494A	p.D4498E
**MUC12**	case 4	7q22.1	100647122	c.A13278C	p.E4426D

AA* means amino acid.

Supplementary Table 8: List of selected candidate genes for network and gene ontology/pathway analysis

CNVs	SNVs	Histone members
TRIM10	MUC4	As shown in Supplementary Table 6 (a total of 64 histone members)
SNHG9	PRB4	
SLC44A4	MUC16	
RXRB	IGFN1	
RPL13P5	PRR21	
PSMB9	PRG4	
NELFE	TAS2R31	
LOC101927526	SPATA31A5, SPATA31A7	
LOC100288162	NBPF10	
CUTA	MUC3A	
CCHCR1	LOC101929983	
APOM	FLG	
NF2	AGAP9	
CHEK2	CACNA1A	
SMARCB1	TRAF7	
BID	MUC12	
BIK	AKT1	
BARD1	WASH1	
BRD1	NF2	
HDAC10	SMARCB1	
IGFBP5	NOTCH2	
MIR6511A2	CHEK2	
MIR6770-2	DRD4	
DAXX	NF1	
NOTCH4	DSPP	
SMARCAL1	DDR1	
KDM4A	N4BP1	
CASP9	BCL11A	
CASP8	KMT2C	
HDAC1	NEDD4L	
KDM1A	TRPM2	
ARID1A	ATF2	
CASP10	ATF6B	
