# Supplementary material for: Whole-genome sequencing identifies new genetic alterations in meningiomas
Source: Oncotarget. 2017 Feb 3;8(10):17070–80. doi: 10.18632/oncotarget.15043 (PMC5370023; doi:10.18632/oncotarget.15043)
Supplement: Supplementary file 2 [file oncotarget-08-17070-s002.doc]

**Table S6. CNVs in Histone members identified in the seven pared Meningioma samples**

| **Case #** | **Chrom^a^** | **Name** | **Haploid** | **Type** | **Confidence** |
| --- | --- | --- | --- | --- | --- |
| **case 1** | chr6 | HIST1H2BL | 2.81 | Gain | high |
| **case 1** | chr1 | HIST2H2BC | 1.55 | Loss | high |
| **case 1** | chr1 | HIST2H3D | 1.55 | Loss | high |
| **case 1** | chr6 | HIST1H3A | 1.5 | Loss | high |
| **case 1** | chr6 | HIST1H2APS1 | 1.63 | Loss | high |
| **case 2** | chr6 | HIST1H4E | 1.44 | Loss | high |
| **case 2** | chr6 | HIST1H2AG | 1.54 | Loss | high |
| **case 3** | chr1 | HIST2H2AC | 2.68 | Gain | high |
| **case 3** | chr6 | HIST1H4A | 2.61 | Gain | high |
| **case 3** | chr6 | HIST1H1B | 2.87 | Gain | high |
| **case 3** | chr6 | HIST1H4J | 2.97 | Gain | high |
| **case 4** | chr6 | HIST1H2BE | 2.73 | Gain | high |
| **case 5** | chr1 | HIST2H3A | 2.77 | Gain | high |
| **case 5** | chr1 | HIST2H3C | 2.77 | Gain | high |
| **case 5** | chr1 | HIST2H4A | 2.82 | Gain | high |
| **case 5** | chr1 | HIST2H4B | 2.82 | Gain | high |
| **case 5** | chr6 | HIST1H2AJ | 2.88 | Gain | high |
| **case 5** | chr6 | HIST1H2AD | 2.71 | Gain | high |
| **case 5** | chr6 | HIST1H2BF | 2.69 | Gain | high |
| **case 6** | chr6 | HIST1H1A | 2.69 | Gain | high |
| **case 6** | chr6 | HIST1H2AL | 2.61 | Gain | high |
| **case 6** | chr6 | HIST1H3I | 2.65 | Gain | high |
| **case 6** | chr6 | HIST1H2AK | 2.71 | Gain | high |
| **case 7** | chr1 | HIST2H2AA3 | 1.2 | Loss | high |
| **case 7** | chr1 | HIST2H2AA4 | 1.19 | Loss | high |
| **case 7** | chr1 | HIST2H2AB | 1.35 | Loss | high |
| **case 7** | chr1 | HIST2H2AC | 1.25 | Loss | high |
| **case 7** | chr1 | HIST2H2BC | 1.29 | Loss | high |
| **case 7** | chr1 | HIST2H2BE | 1.32 | Loss | high |
| **case 7** | chr1 | HIST2H3A | 1.32 | Loss | high |
| **case 7** | chr1 | HIST2H3C | 1.32 | Loss | high |
| **case 7** | chr1 | HIST2H4A | 1.13 | Loss | high |
| **case 7** | chr1 | HIST2H4B | 1.13 | Loss | high |
| **case 7** | chr1 | HIST3H2A | 1.46 | Loss | high |
| **case 7** | chr1 | HIST3H2BB | 1.12 | Loss | high |
| **case 7** | chr1 | HIST3H3 | 1.22 | Loss | high |
| **case 7** | chr6 | HIST1H1A | 1.3 | Loss | high |
| **case 7** | chr6 | HIST1H1B | 1.46 | Loss | high |
| **case 7** | chr6 | HIST1H1C | 1.48 | Loss | high |
| **case 7** | chr6 | HIST1H1D | 1.38 | Loss | high |
| **case 7** | chr6 | HIST1H1E | 1.47 | Loss | high |
| **case 7** | chr6 | HIST1H1T | 1.26 | Loss | high |
| **case 7** | chr6 | HIST1H2AA | 0.96 | Loss | high |
| **case 7** | chr6 | HIST1H2AB | 1.34 | Loss | high |
| **case 7** | chr6 | HIST1H2AC | 1.11 | Loss | high |
| **case 7** | chr6 | HIST1H2AE | 1.27 | Loss | high |
| **case 7** | chr6 | HIST1H2AG | 1.48 | Loss | high |
| **case 7** | chr6 | HIST1H2AH | 1.22 | Loss | high |
| **case 7** | chr6 | HIST1H2AJ | 1.29 | Loss | high |
| **case 7** | chr6 | HIST1H2AK | 1.03 | Loss | high |
| **case 7** | chr6 | HIST1H2AL | 1.48 | Loss | high |
| **case 7** | chr6 | HIST1H2AM | 1.42 | Loss | high |
| **case 7** | chr6 | HIST1H2APS1 | 1.16 | Loss | high |
| **case 7** | chr6 | HIST1H2BA | 1.43 | Loss | high |
| **case 7** | chr6 | HIST1H2BB | 1.46 | Loss | high |
| **case 7** | chr6 | HIST1H2BC | 1.09 | Loss | high |
| **case 7** | chr6 | HIST1H2BE | 1.23 | Loss | high |
| **case 7** | chr6 | HIST1H2BF | 1.38 | Loss | high |
| **case 7** | chr6 | HIST1H2BG | 1.4 | Loss | high |
| **case 7** | chr6 | HIST1H2BI | 1.49 | Loss | high |
| **case 7** | chr6 | HIST1H2BJ | 1.23 | Loss | high |
| **case 7** | chr6 | HIST1H2BL | 1.41 | Loss | high |
| **case 7** | chr6 | HIST1H2BM | 1.19 | Loss | high |
| **case 7** | chr6 | HIST1H2BN | 1.3 | Loss | high |
| **case 7** | chr6 | HIST1H2BO | 1.47 | Loss | high |
| **case 7** | chr6 | HIST1H3A | 1.09 | Loss | high |
| **case 7** | chr6 | HIST1H3B | 1.45 | Loss | high |
| **case 7** | chr6 | HIST1H3C | 1.24 | Loss | high |
| **case 7** | chr6 | HIST1H3E | 1.11 | Loss | high |
| **case 7** | chr6 | HIST1H3F | 1.22 | Loss | high |
| **case 7** | chr6 | HIST1H3G | 1.41 | Loss | high |
| **case 7** | chr6 | HIST1H3H | 1.35 | Loss | high |
| **case 7** | chr6 | HIST1H3I | 1.05 | Loss | high |
| **case 7** | chr6 | HIST1H3J | 1.26 | Loss | high |
| **case 7** | chr6 | HIST1H4B | 1.04 | Loss | high |
| **case 7** | chr6 | HIST1H4C | 1.42 | Loss | high |
| **case 7** | chr6 | HIST1H4D | 1.39 | Loss | high |
| **case 7** | chr6 | HIST1H4E | 1.56 | Loss | high |
| **case 7** | chr6 | HIST1H4F | 1.28 | Loss | high |
| **case 7** | chr6 | HIST1H4G | 1.49 | Loss | high |
| **case 7** | chr6 | HIST1H4H | 1.07 | Loss | high |
| **case 7** | chr6 | HIST1H4I | 1.35 | Loss | high |
| **case 7** | chr6 | HIST1H4J | 1.31 | Loss | high |
| **case 7** | chr6 | HIST1H4K | 1.17 | Loss | high |

Chrom* means Chromosomes.
